# Esophageal Management of an Aortoesophageal Fistula

**DOI:** 10.7759/cureus.86914

**Published:** 2025-06-28

**Authors:** Robert S Gordon, Mrudula Bandaru, Samuel A Schueler, Marie L Borum

**Affiliations:** 1 Internal Medicine, George Washington University, Washington DC, USA; 2 Gastroenterology and Hepatology, George Washington University School of Medicine and Health Sciences, Washington DC, USA; 3 Gastroenterology and Liver Diseases, George Washington University School of Medicine and Health Sciences, Washington DC, USA

**Keywords:** aorta, aortoesophageal fistula, esophageal stent, esophagus, esophagus perforation, tevar complications

## Abstract

Aortoesophageal fistula, the formation of a tract between the aorta and esophagus, is rare and associated with a high mortality rate. We present a 49-year-old woman with an extensive type B aortic dissection found to have a tract between the esophagus and aortic false lumen. Multiple attempts at esophageal stenting were unsuccessful, with subsequent cervical esophagectomy required. Despite the lethal nature of this condition, the gold standard of management is unclear as studies are limited by a low incidence rate.

## Introduction

Aortic dissection is the formation of a false lumen within the aorta. This may occur proximal to the left subclavian artery (LSCA), type A, or distal to the LSCA, type B [1]. Type B aortic dissections (TBAD) have a 65% one-year survival rate with initial management including anti-impulse therapy and thoracic endovascular aortic repair (TEVAR) [1]. Complications of TEVAR include spinal cord ischemia, renal injury, retrograde dissections, endoleaks, and aortoesophageal fistula (AEF) formation [2].

AEFs have a 100% mortality rate in those unable to obtain surgical/procedural intervention [3]. They most commonly result from thoracic aortic aneurysms, post-surgical complications, bone ingestion, and thoracic cancer [3,4]. They can occur from expansion of the aorta resulting in direct contact with the esophagus, leading to pressure necrosis and inflammation and the formation of a fistulous tract [4]. Common strategies for the management of AEF include TEVAR and/or graft placement for management of the aorta and esophageal stenting vs. esophagectomy for management of the esophagus in conjunction with broad-spectrum antibiotics [3].

This report describes the case of a 49-year-old female who presents with an AEF after undergoing TEVAR for the management of a TBAD and aims to highlight the need for further studies to establish standardized management of AEFs and to provide a data point for such studies.

This article was previously presented as a meeting abstract and poster presentation at the 2024 American College of Gastroenterology Annual Meeting on October 27, 2024.

## Case presentation

**Figure 1:**
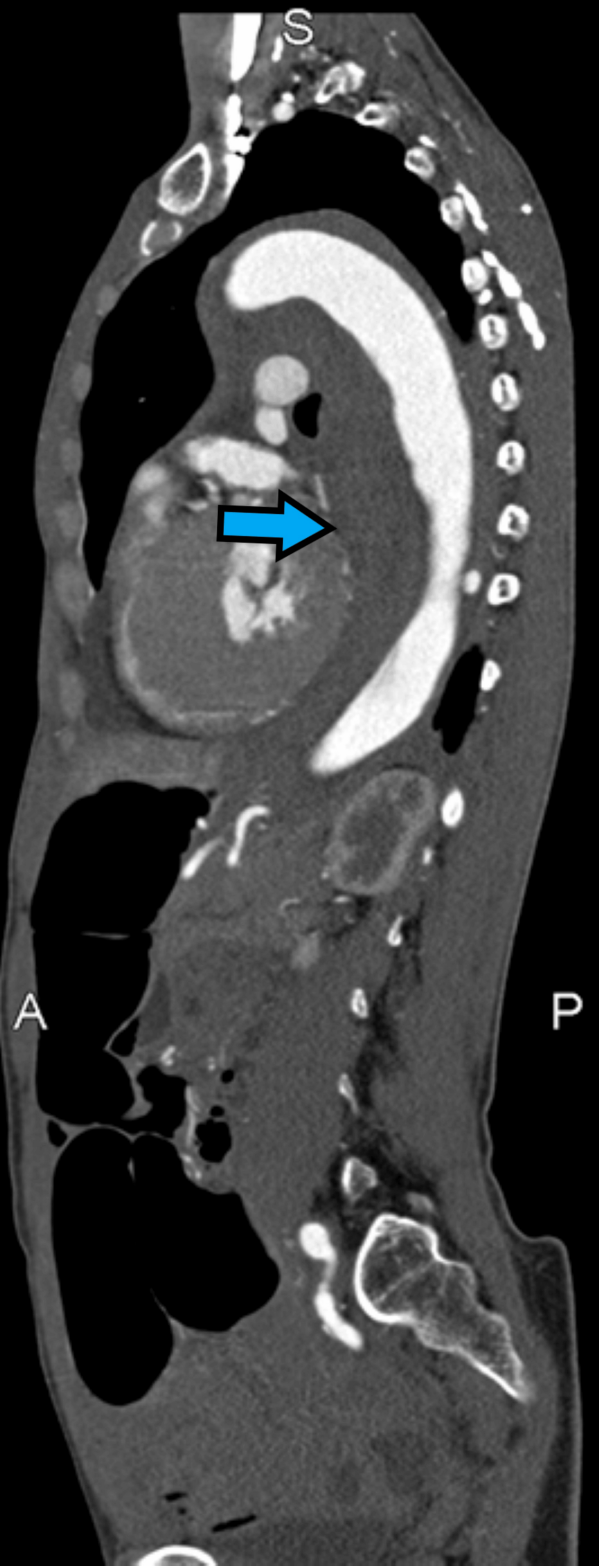
Computed tomography angiogram, sagittal plane, demonstrating a large hematoma within the false lumen of a type B aortic dissection as well as dilated loops of bowel.

**Figure 2:**
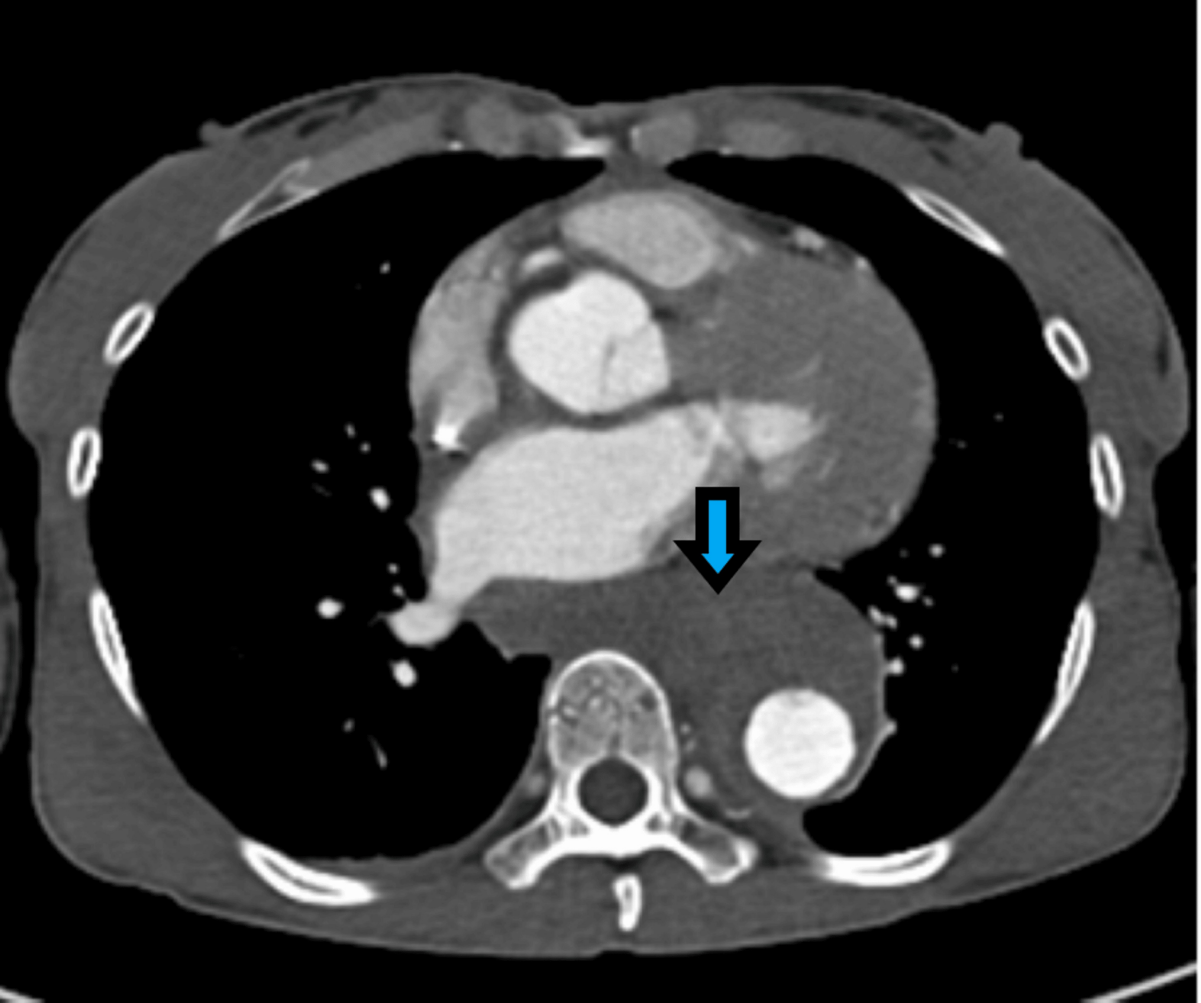
Computed tomography angiogram, axial plane, demonstrating a large hematoma within the false lumen of a type B aortic dissection

A 49-year-old female with hypertension, atrial fibrillation, and cyclic vomiting presented with two days of chest pain radiating to her back. Her initial blood pressure was 213/155 and computed tomography angiogram (CTA) chest demonstrated a TBAD involving the descending aorta extending to the aortic bifurcation, right common iliac, and proximal right external/internal iliac arteries (Figures 1, 2).

**Figure 3:**
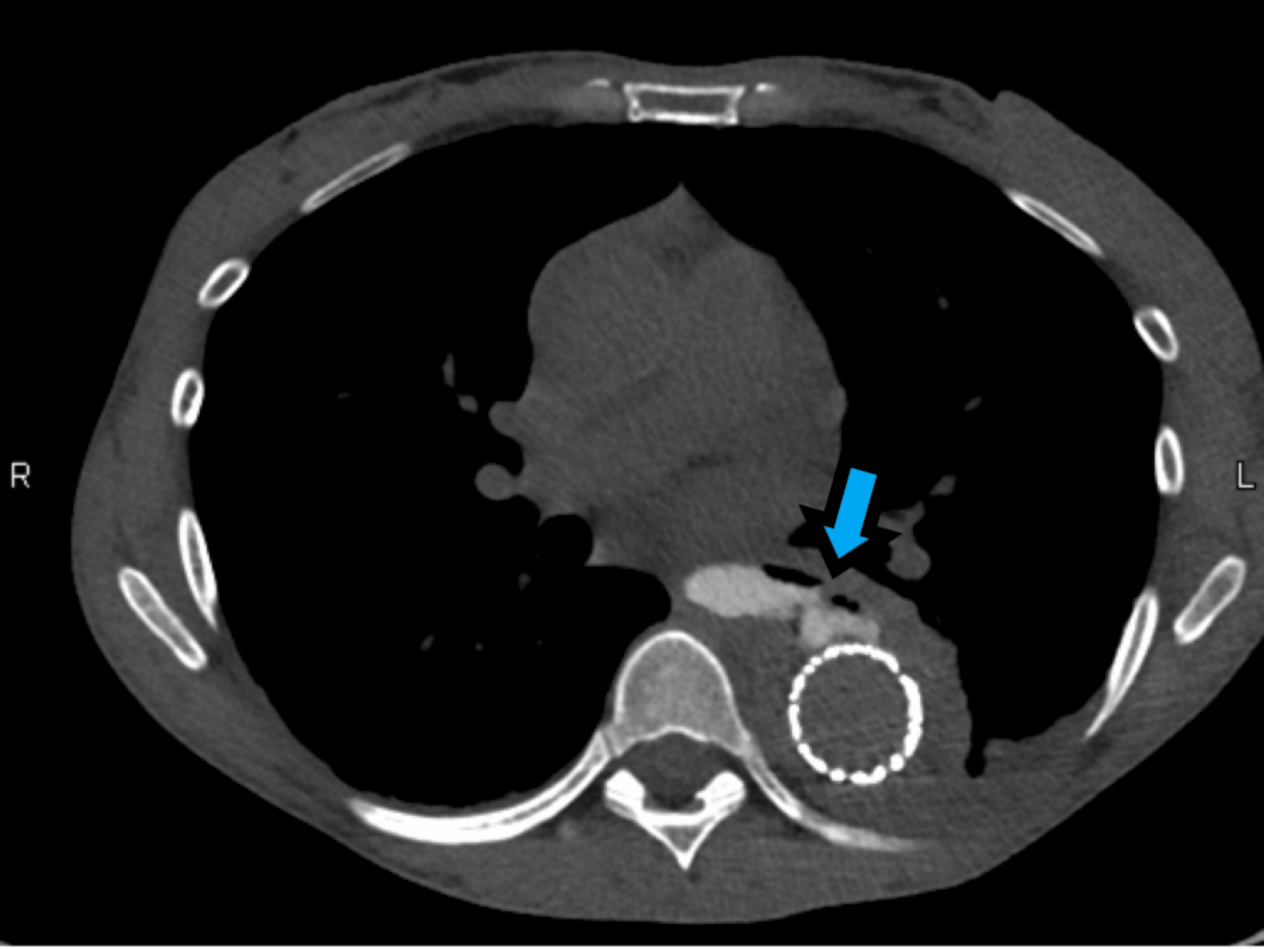
Computed tomography esophagram, axial plane, demonstrating esophageal perforation communicating with the false lumen of the thoracic aorta status post thoracic endovascular aortic repair.

Anti-impulse therapy was initiated, and the patient underwent TEVAR. The post-procedural course was complicated by severe back pain, paralytic ileus, and hematochezia requiring multiple blood transfusions. CTA chest/abdomen/pelvis demonstrated air surrounding the aortic stent in the mid-thoracic aorta, concerning for a fistulous communication between the esophagus and the false lumen of the aorta, subsequently confirmed via CT esophagram (Figure 3).

**Figure 4:**
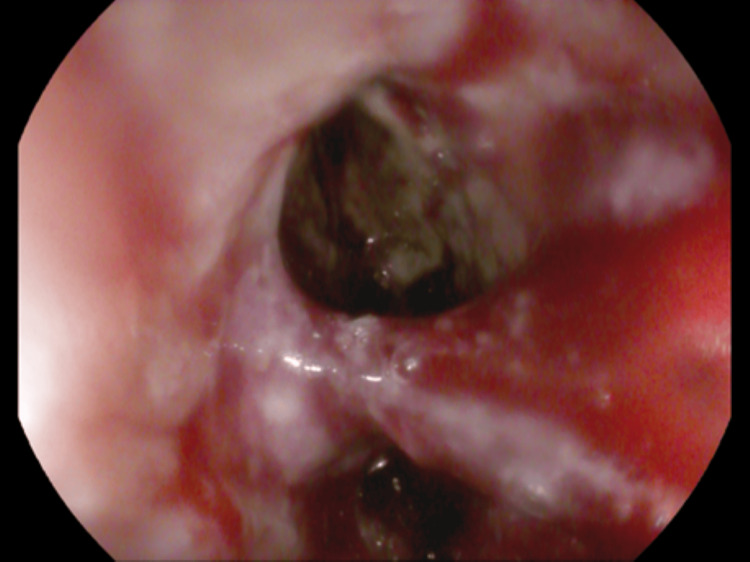
Esophagogastroduodenoscopy image of large esophageal perforation with ulceration at the margins present at 30-33 cm from the incisors with surrounding inflammation.

**Figure 5:**
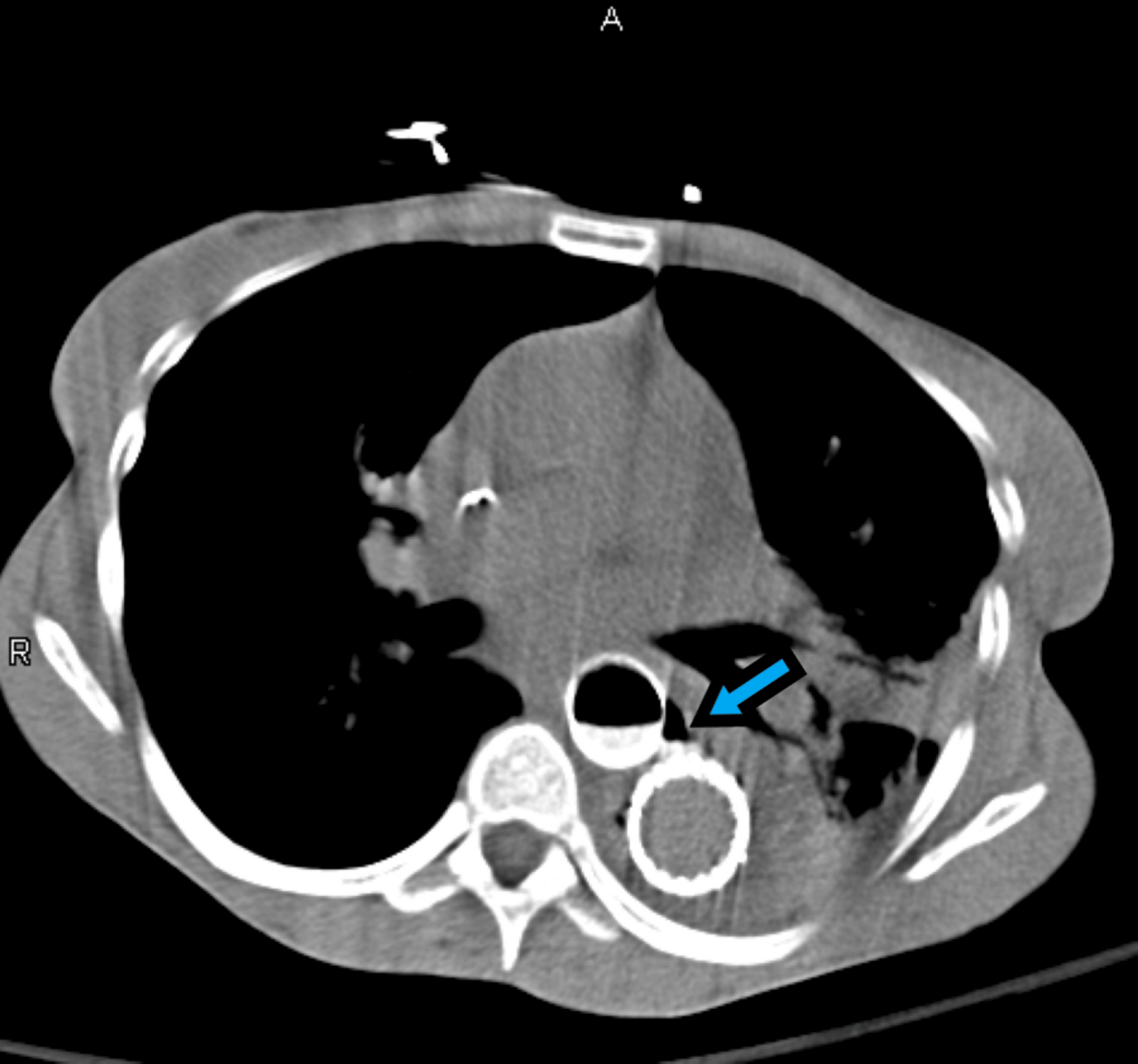
Computed tomography esophagram, axial plane, demonstrating communication between the esophagus and the false lumen of the thoracic aorta status post esophageal stent and thoracic endovascular aortic repair.

Esophagogastroduodenoscopy (EGD) revealed a large esophageal perforation with ulceration at its margins, at 30-33 cm from the incisors, through which the aortic stent was visible (Figure 4). A 23 mm x 155 mm fully covered metal esophageal stent was deployed across the perforation with the proximal end located at 24 cm from the incisors. A subsequent CTA chest with oral contrast demonstrated a persistent leak of oral contrast into the aortic false lumen (Figure 5).

Repeat EGD revealed an intact stent without migration. The stent and clips were endoscopically mobilized off the wall of the esophagus, visualizing an unchanged perforation. Two overlapping self-expanding metal stents (SEMS) were subsequently placed and secured via endo clips. The first stent was deployed across the gastroesophageal junction with the proximal tip at 36 cm from the incisors. The second was placed at 21 cm from the incisors with the distal end overlapping the first stent.

**Figure 6:**
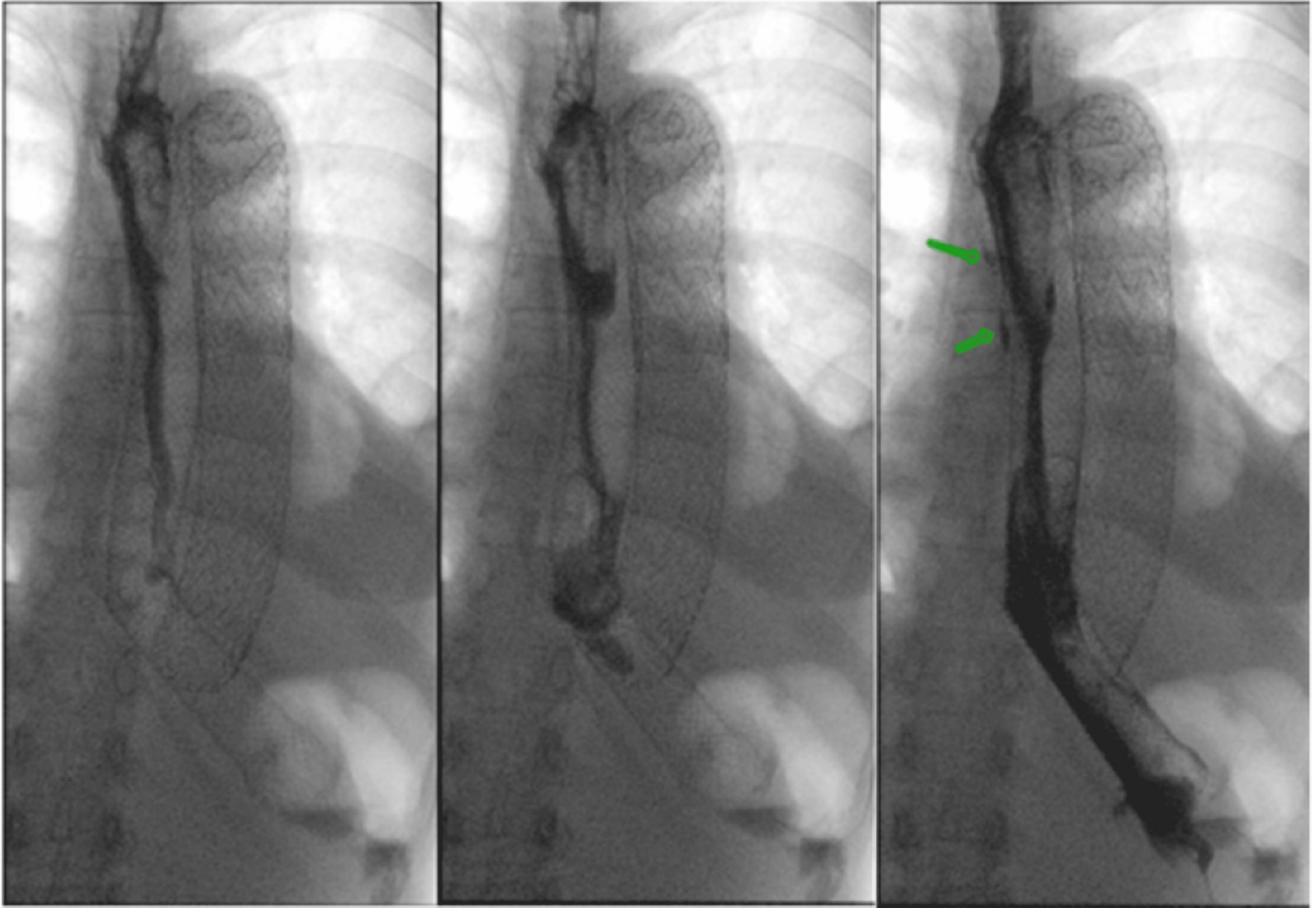
X-ray esophagus with oral contrast status post dual esophageal stent placement demonstrating continued pooling of contrast outside of the lumen of the stent

An esophagram later showed continued contrast leak (Figure 6), resulting in esophageal stent removal, cervical esophagostomy, and placement of a gastrojejunostomy tube. The patient tolerated tube feedings and remained on antibiotics on discharge, with a plan for eventual esophageal reconstruction. The patient returned within 24 hours of discharge in hypertensive emergency with a new, progressively expanding aortic dissection, ultimately resulting in the patient’s death. 

## Discussion

The management of AEFs presents a complex challenge in clinical practice, with conflicting viewpoints in the literature regarding the optimal approach. Advocates for aggressive surgical intervention, such as esophageal resection and aortic graft placement, emphasize the critical need for prompt action to address the life-threatening nature of AEFs [5]. Early surgical intervention, such as esophagectomy, has been shown to reduce the risk of mediastinitis [6]. Notably, patients who undergo esophagectomy have a survival rate of 60.5% [7]. Video-assisted thoracoscopic esophagectomy (VATS-E) is preferred over open thoracic surgery for inducing less postoperative pain and minimizing destruction of the thoracic wall [5]. It also offers a magnified view, allowing safe and accurate dissection of the hard adhesions between the esophagus and aorta in hemodynamically stable patients.

While TEVAR has been recognized as a bridging therapy in hemodynamically unstable patients, the long-term mortality rate of TEVAR performed alone has been reported to be up to 11%, as it does not address the underlying fistula formation, which serves as a conduit for mediastinal infections. Similarly, while esophageal stenting provides an option for early management in patients who are poor surgical candidates, patients who do not undergo an esophagectomy have a poor one-year survival rate [7]. While aggressive surgery offers the best likelihood of overall survival, both esophageal and aortic manipulation have a significant mortality rate ranging from 45.4% to 64% [5].

Yokoe et al. describe a case of a patient with a primary AEF that was successfully managed with VATS-E performed immediately after TEVAR and without aortic replacement. Four months after the TEVAR, esophageal reconstruction was performed with a stomach conduit [6]. In frail patients with high surgical risk, Donato et al. describe a minimally invasive approach that combines staged endovascular and endoscopic treatment. They report three cases of AEF that were successfully managed with the use of TEVAR and the placement of esophageal stents to allow drainage of mediastinal collections. This less invasive technique represents an alternative approach in poor surgical candidates as it is associated with lower intraoperative and perioperative mortality rates [8].

This case demonstrates the difficulty in obtaining a sufficient barrier between the esophagus and mediastinum with esophageal stenting in the setting of an AEF. Despite two attempts at esophageal stenting, repeat imaging displayed a continued leak of esophageal contents after both attempts. This further highlights a likely reason why esophageal stenting alone results in increased incidence of mediastinitis and a poor one-year survival rate compared to esophagectomy, which provides more definitive management. This case provides further evidence that favors esophagectomy over esophageal stenting in the esophageal management of AEF in patients who are surgical candidates. 

## Conclusions

Esophageal stenting provides an initial treatment of patients who are poor surgical candidates; however, an esophagectomy is generally required for definitive management. In surgical candidates, early surgical intervention such as VATS-E reduces the risk of mediastinal infection and increases long-term survival rates. In this case, esophageal stenting was attempted twice with continued leak of contrast noted on esophagram. This highlights how esophageal stenting is insufficient in preventing mediastinal infections when compared to esophagectomy.
